# TriatoKey: a web and mobile tool for biodiversity identification of Brazilian triatomine species

**DOI:** 10.1093/database/bax033

**Published:** 2017-04-17

**Authors:** Luciana Márcia de Oliveira, Raissa Nogueira de Brito, Paul Anderson Souza Guimarães, Rômulo Vitor Mastrângelo Amaro dos Santos, Liléia Gonçalves Diotaiuti, Rita de Cássia Moreira de Souza, Jeronimo Conceição Ruiz

**Affiliations:** 1 Grupo Informática de Biossistemas e Genômica - Centro de Pesquisas René Rachou, Fiocruz Minas. Av. Augusto de Lima, 1715, 30190-002 Belo Horizonte MG -Brazil; 2 Programa de Pós Graduação em Bioinformática - Universidade Federal de Minas Gerais (UFMG), Av. Antônio Carlos 6627 - Pampulha, 31270-901 Belo Horizonte MG -Brazil; 3 Centro Universitário UNA – Instituto de Ciências Biológicas e da Saúde. Rua Guajajaras, 175 Centro, 30180-100 Belo Horizonte MG -Brazil and; 4 Laboratório de Triatomíneos - Centro de Pesquisas René Rachou, Fiocruz Minas, Av. Augusto de Lima, 1715, 30190-002 Belo Horizonte MG -Brazil

## Abstract

Triatomines are blood-sucking insects that transmit the causative agent of Chagas disease, *Trypanosoma cruzi*. Despite being recognized as a difficult task, the correct taxonomic identification of triatomine species is crucial for vector control in Latin America, where the disease is endemic. In this context, we have developed a web and mobile tool based on PostgreSQL database to help healthcare technicians to overcome the difficulties to identify triatomine vectors when the technical expertise is missing. The web and mobile version makes use of real triatomine species pictures and dichotomous key method to support the identification of potential vectors that occur in Brazil. It provides a user example-driven interface with simple language. TriatoKey can also be useful for educational purposes.

**Database URL:** http://triatokey.cpqrr.fiocruz.br

## Introduction

Chagas disease or American Trypanosomiasis is a chronic and potentially fatal infection caused by the protozoan *Trypanosoma cruzi* (1). The parasite is mainly transmitted by feces of blood-sucking infected insects of the subfamily Triatominae (Hemiptera, Reduviidae) (1). Transmission involving the triatomine can be vectorial by direct contact with infected insect feces or orally through food contamination by infected vectors or their feces (2–4). The actual prevalence of Chagas disease is uncertain, but the World Health Organization estimates that ∼6–7 million people are infected worldwide, mostly in Latin America (5). About 4.6 million (95% CI 2.9–7.2 million) of people are estimated to be infected with *T.cruzi* in Brazil (6). Currently, >140 triatomine species are recognized across Latin America, where disease is endemic (7, 8). Sixty-seven triatomine species are found in Brazil (7–11). Some triatomine species can invade human dwellings from natural habitats or even colonize man-made structures, raising the risk of human Chagas disease transmission (12, 13).

Faced with the large number of species with different eco-epidemiological behaviors, the correct taxonomic identification, performed by trained technician staff in municipalities of Brazil, have a major role for the development and improvement of vector control and surveillance strategies. The main method currently used for the triatomine specific identification is the dichotomous key. However, its use requires training and technical expertise. Considering the Oswaldo Cruz Foundation (CPqRR-Fiocruz) engagement in providing human research training for the healthcare technicians, during the training courses we have been observed a great evasion of the trained staff, which consequently leads to a lack of specialists to perform the correct identification in many municipalities of Brazil, resulting in entomological surveillance impairment.

In the context, it is clear the need of interactive, free and easy to use tools that could speed up the entomological surveillance programs.

Here, we have developed the web tool ‘TriatoKey’ (http://triatokey.cpqrr.fiocruz.br), a user-friendly software experience that makes use of pictorial and dichotomous key method. The software development approach employs a series of “yes” or “no” questions conducting the user in direction of the correct species or taxon identification. Basically, the identification is based on morphological characters visualized by photos from catalogued specimens from Chagas Disease Vectors Collection (Fiocruz-COLVEC) (14), as well as, live ones from Fiocruz insectarium colonies.

TriatoKey may be mainly used by healthcare technicians to support routine entomological surveillance when training is missing. It can also be used for taxonomists and for educational activities to improve correct identification and it is available in English, Spanish and Portuguese versions. In addition, a standalone mobile application was developed (available for download at: https://play.google.com/store/apps/details?id=br.fiocruz.cpqrr.triatokey) in order to help such professionals, or non-professionals, that work together with healthcare agencies, in areas where internet is not available by accessing TriatoKey from their personal mobile devices. Another great advantage of such tool is the “geolocation function.” This feature allows users to electronically send georeferenced insect photos that could not be identified by the software to Fiocruz, where taxonomists will be able to help with the correct identification. Finally, this information may help the mapping process of the vectors distribution across the country providing an accurate epidemiological information of triatomine species.

## Methodology

As basic strategy for software development, we used Web technologies such as HTML5, CSS3 and Javascript that gave us access to widely used frameworks and libraries that ease the development of cross-platform apps (15).

Regarding the web application development process, we also adopt the model-view-controller (MVC) pattern approach, where: (i) Model consists of application data, business rules, logic, and functions; (ii) View can be any output representation of information; and (iii) Controller that accepts input and converts it to commands for the Model or View.

For the Controller and Model components, we specially used: (i) AngularJs (http://angularjs.org), (ii) Angular Translate (http://pascalprecht.github.io/angular-translate); and (iii) JSON (http://www.json.org) (16, 17). jQuery (http://jquery.com) and jQuery Mobile (http://jquerymobile.com) were used in order to make the web software accessible on all smartphone, tablet and desktop devices (18). Bower.io (http://bower.io) was used to increase productivity in managing packages and dependencies (19).

To allow sending pictures and GPS coordinate location of possible triatomines captured by users, a standalone version was developed in Android Java language, granting the use of devices like smartphone and tablets with GPS and camera support.

## Database development

**Figure 1.**
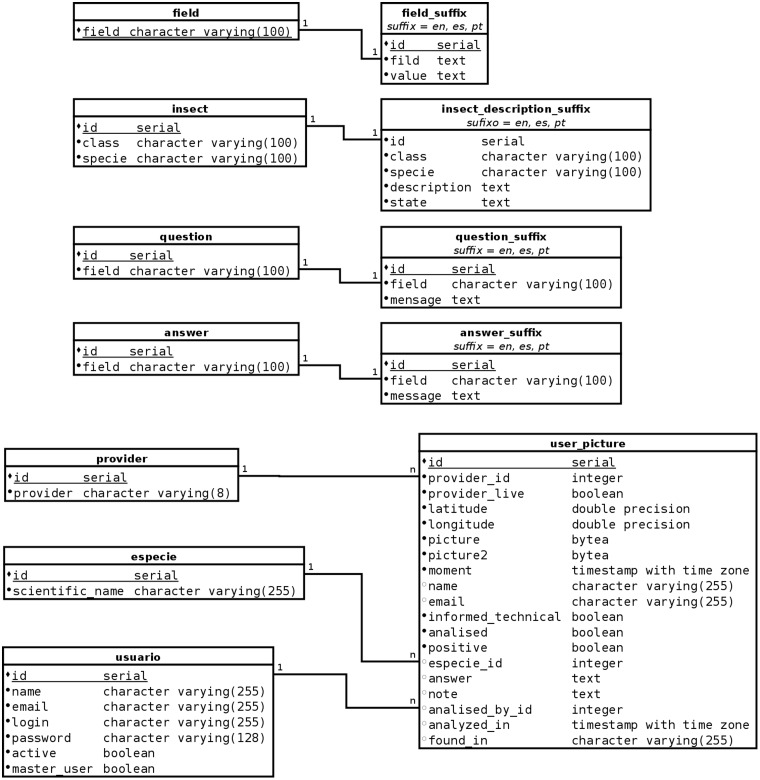
TriatoKey’s relational database schema.

For efficient data and information retrieval, the database linked with TriatoKey was organized into tables containing triatomines records selected by species according morphological characters compiled at Fiocruz-COLVEC. The database was built using PostgreSQL (https://www.postgresql.org) (version 9.4.22), which is an open-source object relational database management system (ORDBMS) (Figure 1). It is important to highlight that the database also includes high-resolution pictures of the insects.

## Database flow

TriatoKey was developed using a multi-tier architecture (often referred as n-tier architecture) or multilayered architecture that could be defined as a client–server architecture in which presentation, application processing and data management functions are physically separated. The multi-tier architecture chose was the three-tier architecture. The first one uses HTML5 and CSS3 together with jQuery and Javascript for the user-friendly application’s interface. The intermediate that controls application’s functionality by performing detailed processing uses PHP (http://php.net) scripts and the last one is the database itself that contains the stored data (Figure 1).

## App Android development

The standalone application (app) for Android OS (Operational System) was developed using Android Studio (version 2.2.2.0) for Linux. Alternatively to the TriatoKey web application, which can be reached from Internet using different web browsers, the standalone app can run without Internet connection making possible the offline use. For taxonomists and health care technicians, this suitability is crucial for a productive fieldwork. Also, the app can be used to photograph possible triatomines and send their images for analysis by FIOCRUZ specialists. When users send pictures, the app gets the current global position by GPS or network and associate this information with the sent pictures. In addition, in order to make the app easily accessible the apk file is also available at Google Play Store (https://play.google.com/store/apps/details?id=br.fiocruz.cpqrr.triatokey).

## Mapping and geolocation technologies

Geolocation measurements for positional estimation were implemented in Triatokey and geographic coordinates together with date and time information are linked to captured images in order to assist the epidemiological surveillance to map the Chagas disease vectors distribution. Regarding the development of data acquisition and analysis processes, a module was created using the Integrated Development Environment (IDE) “Eclipse IDE for Java EE Developers,” Neon version, (https://eclipse.org/) and the framework front-end Materialize, 0.97.98 version (http://materializecss.com/).

The first step of the above-mentioned module involves the photographic capture of the potential bug vector that is subsequently linked to users information (name, e-mail and phone number), the place in the house in which the bug was found, the geographic coordinates, date and time of image capture and transmitted to Fiocruz’s servers.

Immediately after data receiving, an automatic e-mail warning notification in sent to the Fiocruz-Minas specialists and all linked information is submitted to manual curation. At the end of this process, a feedback is sent to users with the final animal identification.

Regarding the geolocation computational approaches, we use: (i) geographical coordinates capture using both a Leaflet library 1.0.2 (http://leafletjs.com/) and OpenStreetMap, an openly licensed map (https://openstreetmap.org/) to report the distribution of the Chagas disease vectors in a color map interface. The markers are grouped by color according to their proximity, zoom level, and incidence using javascript Leaflet Markercluster library, 1.0.0 version (https://github.com/Leaflet/Leaflet.markercluster). The data receiving warning is sent by e-mail using the API Apache Commons Email 1.4 (https://commons.apache.org/proper/commons-email/) in order to optimize the information flow. Colored maps showing the presence of species in a given region can only be visualized by Triatokey administrators and Taxonomists located at Fiocruz Minas.

## Application example

**Figure 2.**
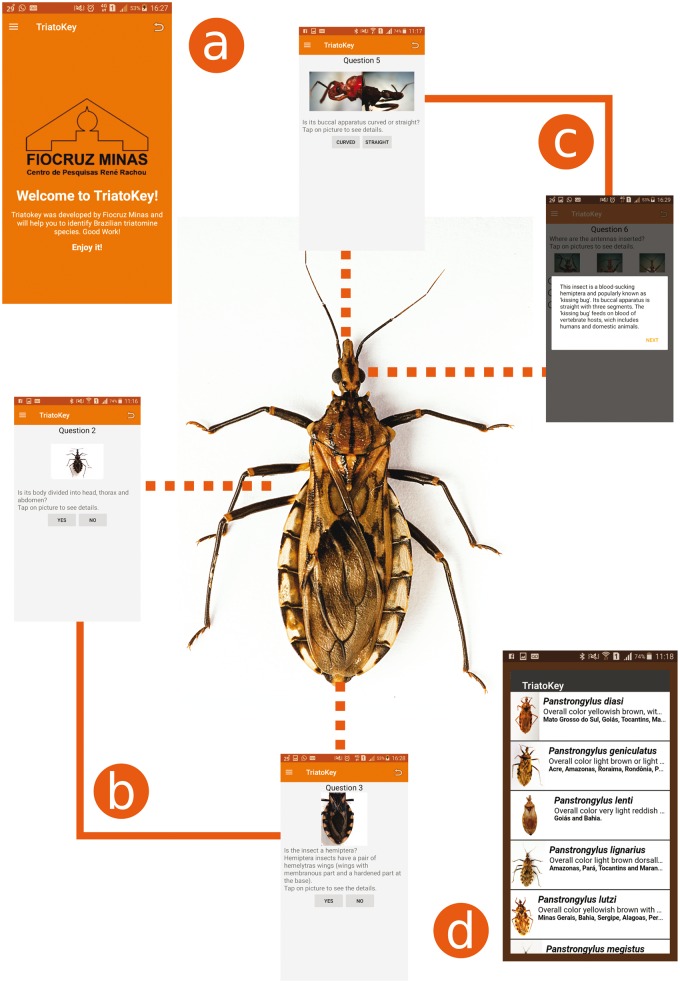
TriatoKey is an interactive tool that employs characteristics of a dichotomous and pictorial key to support the identification of Brazilian species of triatomines (the vectors of *T. cruzi*). In (**a**), the first screen of TriatoKey presenting multi-language options; In (**b**), sequential steps employed in order to identify the bug; In (**c**), key points associated to hematophagous hemiptera and genera identification (throughout of buccal apparatus and morphological location of bug antennae); and in (**d**), final screen of TriatoKey presenting the potential species matching the bug description.

The example illustrated in Figure 2 was built to exemplify a typical use of TriatoKey. Playing a central role in the example is a bug that potentially could transmit Chagas disease. Using the multi-language user-friendly tool, key questions are made to morphological identify the bug as triatomine or not. The crucial elements of the identification process include but are not limited to: (i) verify if the specimen’s features could be associated to class Insecta (Hexapoda); (ii) verify if the bug belongs to order Hemiptera; and (iii) verify specimen’s bucal apparatus and where bug antennae are inserted. At the end of the classification process, the software presents a list including scientific names and pictures of potential candidates that would match the given characteristics.

## Availability

Freely available at http://triatokey.cpqrr.fiocruz.br Standalone version available for download at https://play.google.com/store/apps/details?id=br.fiocruz.cpqrr.triatokey. 
